# Successful pregnancy and weight loss management in a woman unknowingly pregnant at the time of bariatric surgery: a case report

**DOI:** 10.1186/s12884-020-2794-5

**Published:** 2020-02-10

**Authors:** Alireza Khalaj, Fatemeh Ghadimi, Majid Valizadeh, Maryam Barzin

**Affiliations:** 1 Obesity Treatment Center, Department of Surgery, Faculty of Medicine, Shahed University, Tehran, Iran; 2 Obesity Research Center, Research Institute for Endocrine Sciences, Shahid Beheshti University of Medical Sciences, Tehran, Iran

**Keywords:** Bariatric surgery, Pregnancy, Sleeve gastrectomy, Contraception, Obesity

## Abstract

**Background:**

Preventing unintended pregnancy is an important issue for women undergoing bariatric surgery, not only to avoid an adverse fetal outcome but to also ensure maximum weight loss for mother. Current guidelines strongly advise to use a reliable method of contraception following surgery and to delay pregnancy for 12–18 months after surgery.

**Case presentation:**

We present the case of a woman who underwent laparoscopic sleeve gastrectomy while she was unknowingly pregnant. She was monitored closely throughout her pregnancy for maternal-fetal wellbeing and delivered a healthy full-term girl. At her last follow-up visit 6 months post-delivery, both mother and infant were in good general condition and the mother achieved 94.4% excess weight loss.

**Conclusions:**

In all-female patients of childbearing age planning to undergo bariatric surgery, pregnancy should be avoided by using a reliable method of contraception well before surgery. Pregnancy should also be excluded on the day of surgery.

## Background

Obesity is a growing epidemic worldwide, with women slightly affected more than men [1]. Parallel to the increasing prevalence of obesity, there is an increase in bariatric surgeries to achieve sustained weight loss and improvements in several health outcomes. Despite the roughly equal prevalence of obesity, women are disproportionately more likely to utilize bariatric surgery [2]. The increasing popularity of such procedures in women of reproductive age present a significant challenge in the management of future pregnancies. It is currently recommended to postpone pregnancy 12 to 18 months after surgery to lessen the impact of the catabolic and rapid weight loss phase of mother on fetus [3]. The outcomes of pregnancy following bariatric surgery have been significantly investigated, but here we report a case of a woman who underwent laparoscopic sleeve gastrectomy in early pregnancy.

## Case presentation

A 35-year-old female with initial weight of 103.4 kg and body mass index of 35.8 kg/m^2^ was considered for bariatric surgery due to significant impairment of quality of life and failure of intensive lifestyle modification to maintain weight loss for 1 year. Her past medical history was notable for hypothyroidism, which was controlled with 0.5 mg of Levothyroxine daily. She did not have diabetes, hypertension, or any other obesity-related co-morbidities except fatty liver grade 1 with an increased liver span of 18.3 cm found on abdominal ultrasonography. Nutritional evaluation, including micronutrient measurements, was unremarkable.

Her menstrual cycles had been regular and she had no history of infertility. Her obstetric history was significant for one full-term pregnancy ended by an uncomplicated elective cesarean section 7 years ago and one first-trimester spontaneous abortion 6 months ago. She was also found to have a negative serum β-hCG level 1 week before surgery.

She underwent successful and uncomplicated laparoscopic sleeve gastrectomy, creating a gastric tube over a 36-F bougie with the exclusion of 80% of the stomach, and was discharged on postoperative day two. She missed her first menses on the third week following surgery and was found to have positive serum β-hCG levels at that time. She discontinued oral hormonal contraceptives 3 months before surgery for personal reasons and her last menstrual period was precisely 11 days before surgery. Retrospectively, she acknowledged a history of unprotected sexual intercourse 3 days before surgery for which she took a dose of 1 mg of Levonorgestrel + Ethinyl Estradiol 100 mcg (2 pills of Contraceptive HD/Ovocept-HD) within 1 h after intercourse and then repeated it after 12 h. Two months later an intrauterine gestation of approximately 11 weeks (CRL = 41 mm) was identified by an obstetric ultrasound. Although adequate counseling was provided for the couple regarding the potential adverse effects of surgery and rapid weight loss phase on pregnancy outcome, they decided to continue with the pregnancy.

**Fig. 1:**
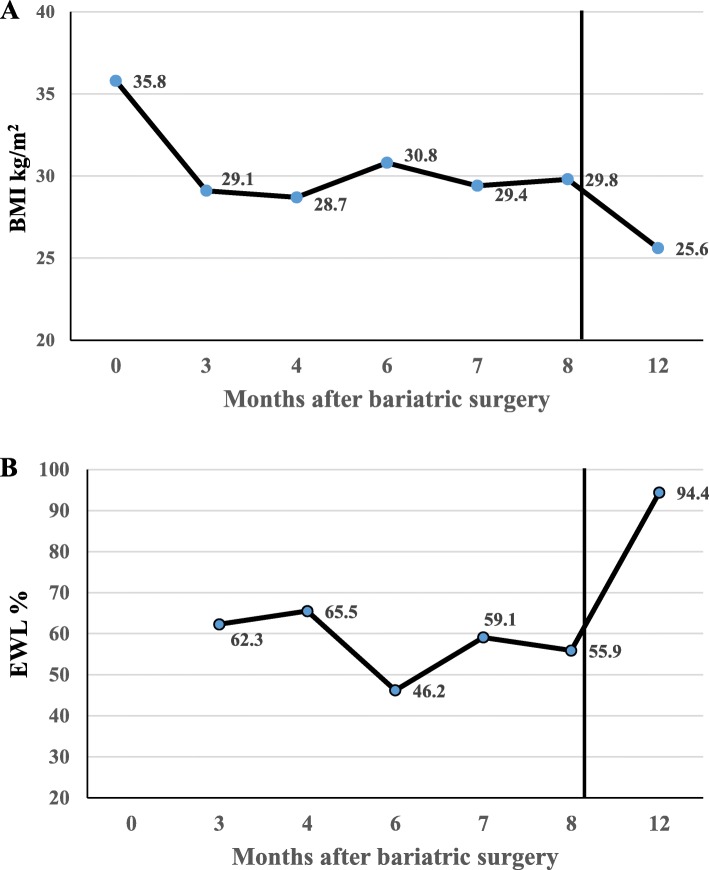
Body mass index (BMI) (**a**) and excess weight loss (EWL%) (**b**) before and 12 months after bariatric surgery. Vertical line indicates time of delivery

She was monitored throughout her pregnancy for proper weight gain, nutritional supplementation and surveillance, and for fetal wellbeing by a multidisciplinary team including high-risk obstetrics, bariatric surgeons, and nutritionists. She was also prescribed prenatal vitamins Naturemade® tablets daily (containing 27 mg ferrous fumarate, 11 mg zinc, 800 mcg folic acid, 5.2 mcg vitamin B12, vitamins A, B group, C, D, E, K, and biotin) and one Calcicare tablet daily (200 IU vitamin D, 400 mg calcium, 100 mg magnesium, and 4 mg zinc) up to the end of pregnancy. Her pregnancy continued uneventfully, and she underwent an elective cesarean section at 39-weeks to deliver a healthy full-term girl weighing 2900 g (between 10th and 25th percentile), and 51 cm in length with a head circumference of 33 cm. The infant reached all expected milestones during the first 6 months of her life, and the mother achieved 94.4% excess body weight loss. Her BMI at 12 months post-surgery were 25.6 kg/m^2^ (Fig. 1).

## Discussion and conclusions

Bariatric surgery is considered an effective intervention for lasting weight loss but reports have been inconsistent with its impact on pregnancy outcomes. There is a need for explicit perioperative instructions regarding fertility, pregnancy, and contraception with the increasing number of women of reproductive age utilizing bariatric surgery. Based on several systematic reviews and meta-analysis, there is a consensus that bariatric surgery resulted in a reduced risk of gestational diabetes mellitus, hypertensive disorders, large for gestational age, and macrosomia in subsequent pregnancies. However, the odds of preterm delivery, small for gestational age infants, and intrauterine growth restriction were increased [4–6].

There have been reports of adverse maternal and fetal outcomes for pregnancies occurring within 1 year of surgery such as maternal internal herniation and consequent bowel obstruction [7–10], intussusception [11], volvulus [12], maternal micronutrient deficiencies [13], fetal neural tube defects [14], and chondrodysplasia punctata [15].

The literature also contains two other case reports of early-stage pregnancy at the time of bariatric surgery. Mavor et al. reported a 26-year-old woman with an intensive weight loss prior to sleeve gastrectomy who was discovered to be pregnant at the time of surgery. Her pregnancy ended with a healthy newborn, but she herself failed to achieve a healthy BMI throughout 24 months of follow up [16]. A second case, reported by Rye et al., involved a 33-year-old woman who underwent 6 weeks of liquid meal replacement preoperatively, and then laparoscopic Roux-en Y gastric bypass. She experienced upper GI symptoms following surgery, and it was found accidentally on abdominal ultrasound that she was pregnant. She decided to undergo elective termination of pregnancy [17]. Both cases indicated pregnancy as a result of increased fertility from preoperative weight loss. However, our case report illustrates pre-operative contraception failure.

Current guidelines recommend delaying pregnancy for up to 18 months after surgery but there is no compelling evidence that pregnancy during the first postoperative year might adversely affect the outcomes. However, preventing unintended pregnancy is significant in the perioperative period because it is a weight dilemma: gaining weight to support adequate growth and development of the fetus which contradicts the goals of the surgery and ultimately may alter the weight loss achieved.

The current guideline emphasizes counseling for contraceptive use following surgery, but preoperative contraception recommendation in order to avoid pregnancy at the time of surgery is neglected. It seems reasonable to recommend starting a contraceptive method prior to surgery and continuing with it after surgery. Although there are no quality data exists to help guide appropriate contraceptive choice, any contraception method is more efficacious and safer than no method at all. The choice of contraception for these patients requires careful consideration to balance the risk of decreased efficacy associated with obesity and the type of bariatric surgery against the individual needs and preferences [18].

In theory, bariatric surgery may affect the efficacy of oral contraceptives by altering the absorptive capacity of the gut. Dietary restrictions, as well as vomiting and diarrhea for the first few weeks postoperatively may even further reduce the effectiveness of oral contraceptives. Thus, non-oral methods of contraception, including long-acting reversible contraception methods (intrauterine devices and implants) should be considered and offered when women receiving counselling regarding bariatric surgery [19].

In addition to initiating an appropriate method of contraception well before surgery, a routine pregnancy test for all patients of reproductive age should be performed on the day of surgery, but a very early gestation cannot be identified by commonly used pregnancy tests. To eliminate cases like ours, we suggest delaying surgery until pregnancy can be definitively excluded, for instance, by taking a thorough sexual history. In the case of pregnancy at the time of or following surgery, women should receive detailed information about maternal and fetal risks such as preterm delivery, small for gestational age infants, and intrauterine growth restriction as previously discussed. If a woman desires to maintain her pregnancy, she should be followed closely throughout her pregnancy to assess weight changes, nutritional deficiencies, and fetal health to ensure optimal pregnancy outcomes.

In conclusion, to prevent unintended pregnancy pre-operatively, patients of reproductive age should be provided appropriate referrals and counseled by a family planning physician on each contraceptive method’s risks, benefits and alternatives and have a plan in place prior to surgery. Pregnancy should be excluded confidently on the day of surgery using a reliable and sensitive test. In the case of pregnancy and a woman’s desire to continue it, close monitoring of maternal-fetal wellbeing is necessary.

## Data Availability

The data referred to in this case report was obtained from review of the patient’s medical and surgical record and is not publicly available.

## Abbreviations

BMI: Body mass index
CRL: Crown-rump length
EWL: Excess weight loss
GI: Gastrointestinal
β-hCG: Beta-human chorionic gonadotropin
